# Direct targeting sperm-associated antigen 9 by miR-141 influences hepatocellular carcinoma cell growth and metastasis via JNK pathway

**DOI:** 10.1186/s13046-016-0289-z

**Published:** 2016-01-21

**Authors:** Guohua Lou, Xuejun Dong, Caixia Xia, Bingjue Ye, Qiuyue Yan, Shanshan Wu, Ye Yu, Feifei Liu, Min Zheng, Zhi Chen, Yanning Liu

**Affiliations:** State Key Laboratory for Diagnosis and Treatment of Infectious Diseases, Collaborative Innovation Center for Diagnosis and Treatment of Infectious Diseases, The First Affiliated Hospital, College of Medicine, Zhejiang University, 79# Qingchun Road, 6A-17, Hangzhou, 310003 China; Shaoxing People’s Hospital, Shaoxing Hospital, Zhejiang University, 79# Qingchun Road, 6A-17, Hangzhou, 310003 China

**Keywords:** SPAG9, HCC, miR-141, JNK

## Abstract

**Background:**

The aberrant expression of sperm-associated antigen 9 (SPAG9) is associated with numerous cancers, including hepatocellular carcinoma (HCC). The exploration of molecules and mechanisms regulating SPAG9 expression may provide new options for HCC therapy.

**Methods:**

MiRNA target prediction programs were used to explore SPAG9-targeted miRNAs. SPAG9 and miR-141 expression were detected in HCC tissues and cell lines by Western blot and real-time PCR. Dual-luciferase reporter assay was utilized to validate SPAG9 as a direct target gene of miR-141. Cell proliferation, invasion, and migration assays were used to determine whether miR-141-mediated regulation of SPAG9 could affect HCC progression.

**Results:**

An inverse correlation was observed between SPAG9 and miR-141 expression in HCC tissues and cell lines. Dual-luciferase reporter assay further showed that SPAG9 was a direct target gene of miR-141. The ectopic expression of miR-141 could markedly suppress SPAG9 expression in HCC cells. MiR-141 overexpression also resulted in significantly reduced cell proliferation, invasion, and migration, and imitation of the SPAG9 knockdown effects on HCC cells. Furthermore, SPAG9 restoration in miR-141-expressing cells sufficiently attenuated the tumor-suppressive effects of miR-141. Finally, JNK activity was found to be reduced by miR-141 overexpression the same way as by SPAG9 silencing. The overexpression of SPAG9 lacking its 3′-UTR significantly restored JNK activity and its downstream genes in miR-141-transfected HCC cells.

**Conclusion:**

MiR-141 suppression may cause aberrant expression of SPAG9 and promote HCC tumorigenesis via JNK pathway.

**Electronic supplementary material:**

The online version of this article (doi:10.1186/s13046-016-0289-z) contains supplementary material, which is available to authorized users.

## Background

Hepatocellular carcinoma (HCC) is the third cause of cancer-related mortality in the world [1]. Like other cancers, HCC is the result of a complex process associated with various genetic and epigenetic changes acting through etiology-specific pathways [2]. However, the complicated molecular pathogenesis of HCC remains poorly understood, and the long-term survival rate continues to be low over the past two decades, despite existing strategies for HCC treatment. Therefore, further uncovering the molecular mechanisms of HCC and exploring new therapeutic targets to improve HCC treatment are very important.

Sperm-associated antigen 9 (SPAG9), which is a new member of the cancer testis (CT) antigen family, was involved in a c-Jun NH2-terminal kinase (JNK) signaling pathway [3, 4]. Several studies have reported an association of aberrant SPAG9 expressions in various types of human cancers including breast, thyroid, cervical, and colon carcinoma [5–8]. The down-regulation of SPAG9 by siRNA approach could also inhibit tumor cell proliferation and invasion [9, 10]. Recently, SPAG9 overexpression was identified to be correlated with poor prognosis and tumor progression in human HCC [11]. However, the underlying mechanism causing SPAG9 overexpression in HCC remains unclear.

MicroRNAs (miRNAs) are small non-coding RNAs that can cause mRNA degradation or translation inhibition by interacting with the 3′-untranslated region (3′-UTR) of the target gene mRNA [12–14]. Accumulated evidences have shown that miRNAs play crucial roles in HCC development through regulating the expression of oncogenes or tumor suppressor genes [15, 16]. An aberrant expression of miRNAs such as miR-122, miR-184, miR-106b, miR-219, miR-31, and miR-362-5p has been reported to regulate tumor cell growth, apoptosis, migration, and invasion by targeting proteins involved in those cellular pathways [17–22]. However, certain miRNA, which can target and regulate the expression of SPAG9, has not been identified.

In the present study, we used miRNA target prediction programs to explore SPAG9-targeted miRNAs in hepatocarcinogenesis and identified miR-141 as an endogenous regulator of SPAG9 in HCC. MiR-141-mediated SPAG9 regulation was also found to play important roles in HCC cells growth, invasion, and migration. The data in the present study suggest that suppression of miR-141 may cause an aberrant overexpression of SPAG9 and that miR-141-mediated SPAG9 regulation may be a potential strategy for HCC therapy.

## Methods

### Patients and clinical tissue specimens

Matched fresh HCC specimens and non-tumorous liver samples were obtained from 10 clinically confirmed HCC patients during hepatic resection at the First Affiliated Hospital of Zhejiang University. Samples were either immediately snap-frozen in liquid nitrogen. The hospital’s committee of ethics approved this study, and informed consent was obtained from all patients.

### Cell lines and cell culture

Human HCC cell lines (HepG2, Huh7, LM3 and Hep3B), the human immortalized liver cell line HL-7702, and the human embryonic kidney cell line HEK293T were purchased from ATCC (American type culture collection). These cells were maintained in their complete growth medium according to the culture method.

### Isolation and detection of miRNA

Total RNA enriched with miRNAs was isolated from HCC samples or HCC cells by using miRVana miRNA isolation kit. Then real-time PCR analysis was performed to examine miR-141 expression according to the manufacturer’s instructions (Ambion Diagnostics, TX). Data are normalized over the average CT value of U6, and 2^-ΔΔCT^ method was used to determine relative miRNA expression.

### Western blotting

HCC samples or HCC cells were lysed with RIPA peptide lysis buffer (Beyotime Biotechnology, Jiangsu, China) containing 1 % protease inhibitors (Pierce) and Western blotting analysis were performed according to standard procedures. Primary antibodies were used as follows: anti-SPAG9 (1:1000, Abcam), anti-JNK, anti-p-JNK, anti-c-Jun, and anti-MMP9 (1:2000, Abcam) and anti-GAPDH (1:3000, Huabio). Protein bands were developed using the Enhanced Chemiluminescence (ECL) system and were visualized and quantified by using the ChemiScope Western Blot Imaging System (Clinx Science Instruments Co., Ltd).

### siRNA, miRNA and plasmid transfection

SiRNA against SPAG9 and scrambled siRNA were purchased from RiboBio, mirVana™ negative control and mirVana™ miR-141 mimics were purchased from Life Technologies Inc, and SPAG9 overexpression plasmid were constructed by Genechem. SiRNA and plasmid transfection was carried out using lipofectamine™ 2000 (Invitrogen), miRNA transfection was carried out using lipofectamine™ RNAiMAX (Invitrogen) according to the manufacturer’s instructions. At 24 h after transfection, the effects of gene silencing and miR-141 expression were respectively measured by western blot analysis and real-time PCR analysis.

### Dual-luciferase assay

The 3′-UTR of SPAG9 containing the potential binding sites of miR-141 was amplified using the following primers: 5′- GGCGGCTCGAGAAAATCCGTTCTACCATAAC -3′, and 5′- AATGCGGCCGCAACTCAATCAACATCACCAT -3′. The PCR products were inserted into pmiR-RB-REPORT™ vector within XhoI/NotI restriction sites. Mutation was performed using a fast mutation kit (NEB, Canada). HEK293T cells were co-transfected with wild-type or mutant 3′-UTR of SPAG9 and miR-141 or the control mimics. After 48 h, the cells were lysed, and the firefly and Renilla luciferase activities were measured with the Dual-Luciferase Reporter Assay System (Promega). The results are presented as the ratio of Renilla luciferase activity to firefly luciferase activity.

### Cell proliferation assay

At 24 h after transfection with SPAG9 siRNA or miR-141 mimics, HCC cells were plated in 96-well plates at approximately 5,000 cells per well and maintained in culture medium for 72 h. Cell proliferation was determined by methylthiazolyldiphenyl-tetrazolium bromide (MTT) assay (Sigma).

### Wound-healing assay

At 24 h after transfection, HCC cells were plated in 6-well plates at approximately 1 × 10^6^ cells per well. At 100 % confluency, cells were scratched with a 200-uL filter tip to create an artificial wound. After wounding, the medium was changed to fresh serum free medium to remove cellular debris. Serial images were obtained at 0, 24 and 48 h.

### Cell invasion assays

Cell invasion was assessed using the Matrigel Invasion Chamber (BD Biosciences). Cells (1 × 10^5^) were seeded on transwell chambers with Matrigel in serumfree medium. Medium containing 10 % FBS in the lower chamber served as the chemo-attractant. The invasive cells that attached to the lower surface of the membrane insert were fixed with 4 % paraformaldehyde, stained with 1 % crystal violet, and counted. Each experiment was carried out in triplicate in 3 independent experiments to ensure consistency.

### RNA isolation and real-time PCR analysis

Total RNA was isolated from HCC cells with TRIzol (Invitrogen), real-time PCR was performed on ABI 7900 system (Applied Biosystems) using SYBR Premix EX TaqTM II kit (Takara) to examine the expression of MMP9 and c-Jun, GAPDH was used as internal control. The 2^-ΔΔCT^ method was used to determine relative mRNA expression.

### Statistical analysis

Differences between groups were analyzed using conventional Student’s t test or ANOVA. Each experiment was repeated at least three times, and the data are presented as mean ± SD. A *P*-value of 0.05 or less was considered as statistically significant.

## Results

### SPAG9 expression level is inversely correlated with miR-141 in HCC

**Fig. 1:**
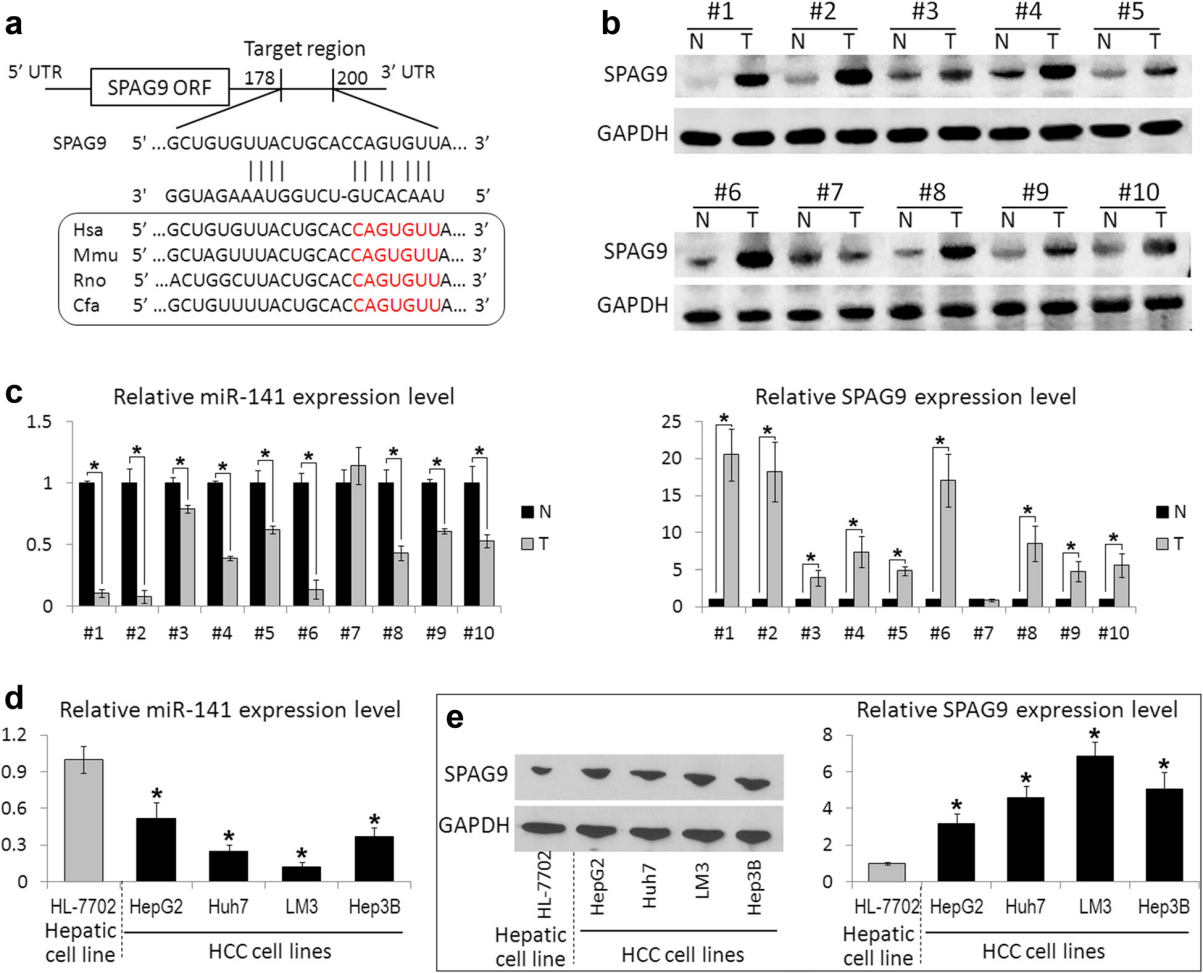
SPAG9 expression is inversely correlated with miR-141 level in HCC. **a** The location of target sites of miR-141 in the SPAG9 3′-UTR is shown. **b** The protein level of SPAG9 in 10 paired HCCs (T) and non-cancerous hepatic tissues (N) were determined by Western blot analysis. (C and D) The expression levels of miR-141 were performed by real time-PCR in HCC tissues (**c**) and cell lines (**d**). **e** Western blot analysis of SPAG9 expression levels in human liver cell lines. Data are presented as the mean ± S.D. (*P < 0.05, n = 3)

By using three publicly available algorithms (TargetScan, miRanda, and PicTar), miR-141 was identified as a candidate miRNA that could target SPAG9. As shown in Fig. 1a, miR-141 has potential target sites in the 3′-UTR of the SPAG9 mRNA sequence. Moreover, the miR-141 binding sequences in the SPAG9 3′-UTR are highly conserved across species.

We then examined the expression levels of miR-141 and SPAG9 in 10 randomly selected HCC tissues paired to adjacent non-cancerous liver tissues. As we expected, 9 out of 10 HCCs (90 %) had increased SPAG9 expression as compared with corresponding non-cancerous hepatic tissues (Fig. 1b); whereas, the expression level of miR-141 was decreased in these 9 HCC tissues as compared with corresponding non-cancerous tissues (Fig. 1c). Furthermore, the miR-141 and SPAG9 expressions were observed in four human HCC cell lines (HepG2, Huh7, LM3, and Hep3B) and an immortalized liver cell line HL-7702. Consistent with the data obtained from HCC tissues, the expression patterns of SPAG9 were also inversely correlated with those of miR-141 in cell lines (Fig. 1d and e).

### SPAG9 is the direct downstream target of miR-141

**Fig. 2:**
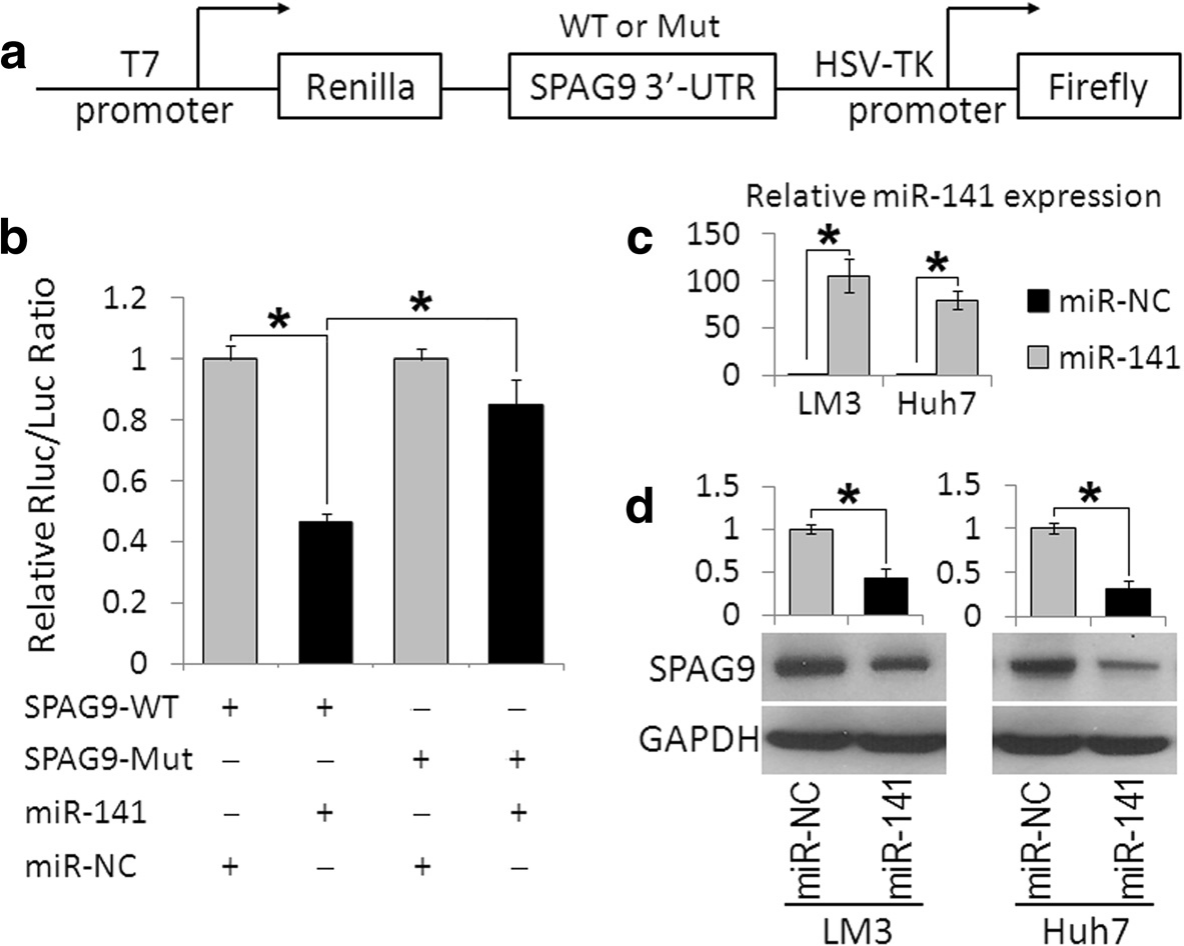
Validation of SPAG9 as the direct target of miR-141. **a** Wild-type (WT) and mutant (Mut) 3′-UTR of SPAG9 were cloned into a pmiR-RB-REPORT™ vector. **b** Dual-luciferase activity of the wild-type (WT) and mutant (Mut) SPAG9 3′-UTR reporter constructs in the presence of miR-141 or control miRNA (miR-NC). **c** MiR-141 expression levels were analyzed by real time-PCR in miR-141- or control miRNA- (miR-NC) transfected LM3 and Huh7 cells. **d** Western blot analysis of SPAG9 expression in LM3 and Huh7 cells transfected with miR-141 mimics or control miRNA. Data are presented as the mean ± S.D. (*P < 0.05, n = 3)

To determine whether SPAG9 was a direct target of miR-141 in HCC, we cloned 728 bp fragments of the human SPAG9 3′-UTR mRNA containing the putative miRNA-binding site into the pmiR-RB-REPORT™ vector (Fig. 2a) and transfected this constructed vector (SPAG9-WT) into HEK293T cells along with miR-141 mimics, or a non-target control miRNA (NC). Compared with miR-NC, miR-141 induced a significant decrease in the normalized luciferase activity of the vector containing the putative miRNA-binding site. In addition, the mutation of the miR-141-responsive elements in the binding site of SPAG9 3′-UTR (SPAG9-Mut) resulted in an abrogation of the inhibitory effects of miR-141 (Fig. 2b).

The correlation between miR-141 and SPAG9 was further examined by evaluating the expression of SPAG9 in the human HCC cell line Huh7 and LM3 after the overexpression of miR-141. As anticipated, the overexpression of miR-141 by transfection with miR-141 mimics significantly reduced SPAG9 protein levels in Huh7 and LM3 cells (Fig. 2c and d). These data strongly suggest that SPAG9 is a direct target of miR-141. The aberrant overexpression of SPAG9 in HCC may be caused by the reduced expression of miR-141.

### MiR-141-mediated downregulation of SPAG9 inhibits HCC cell growth, migration, and invasion

**Fig. 3:**
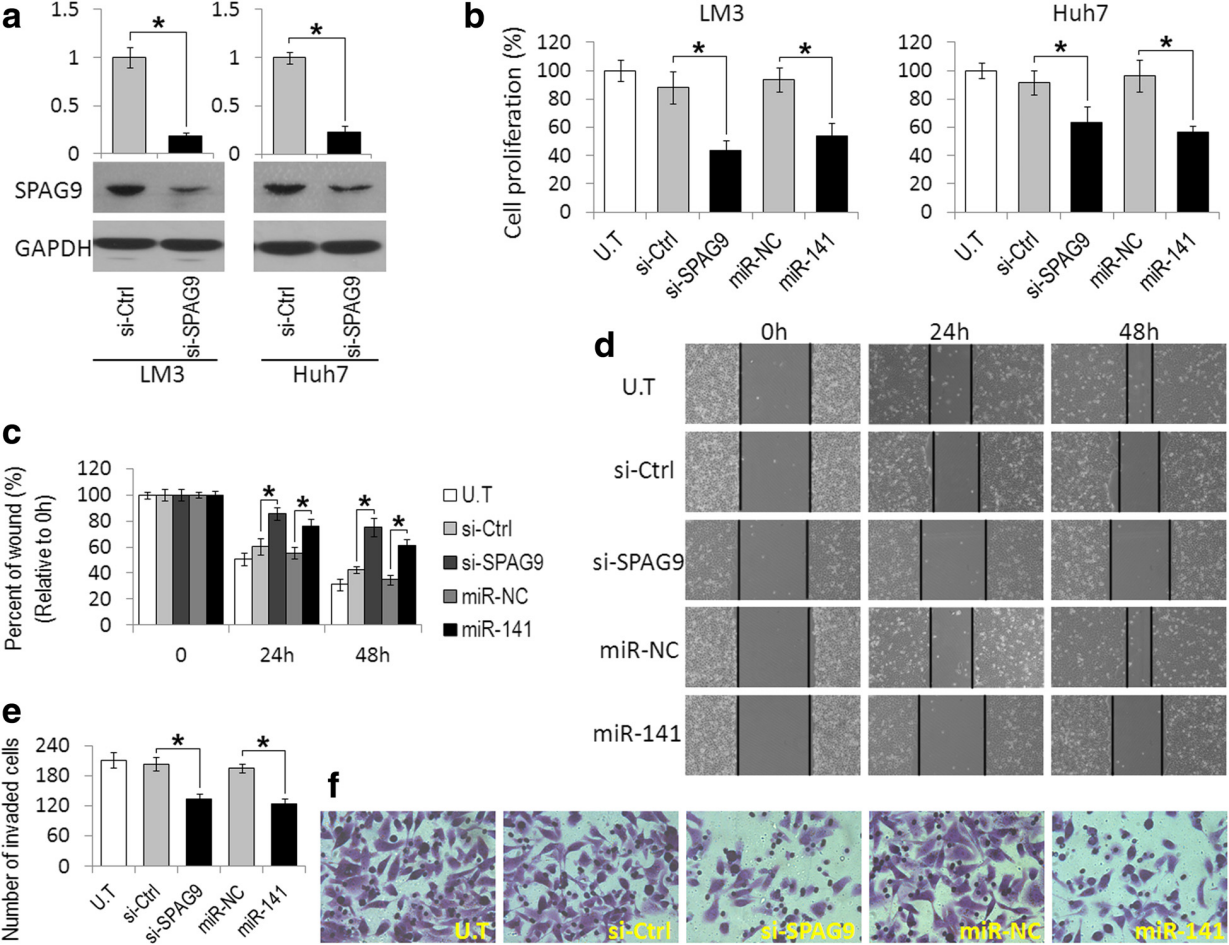
Inhibition of SPAG9 or upregulation of miR-141 reduces proliferation, migration, and invasion of HCC cells. **a** Western blot analysis of SPAG9 expression in LM3 and Huh7 cells transfected with SPAG9 siRNA (si-SPAG9) or control siRNA (si-Ctrl). **b** MTT assay showed that the inhibition of SPAG9 or ectopic expression of miR-141 could hinder Hep3B and Huh7 cell proliferation. **c** and **d** Wound-healing assay showed that SPAG9 depletion or miR-141 upregulation decreased cell migration of Huh7 cells. **e** and **f** Matrigel invasion assay showed that SPAG9 depletion or miR-141 upregulation decreased cell invasion of Huh7 cells. Data are presented as the mean ± S.D. (*P < 0.05, n = 3). U.T: untreated Huh7 cells

The above-mentioned results prompted us to further explore the functional relationship between miR-141 and SPAG9. MTT assay manifested that the downregulation of SPAG9 by transfection with SPAG9 siRNA in Huh7 and LM3 cells resulted in a significant suppression of cell proliferation (Fig. 3b). Likewise, a substantial reduction of cell proliferation was observed in miR-141 mimic-transfected cells (Fig. 3b). In addition, wound-healing and transwell assays showed that Huh7 cells transfected with SPAG9 siRNA or miR-141 mimics displayed reduced migratory and invasive activity as compared with control siRNA (si-Ctrl) or miR-NC transfected cells (Fig. 3c-f).

**Fig. 4:**
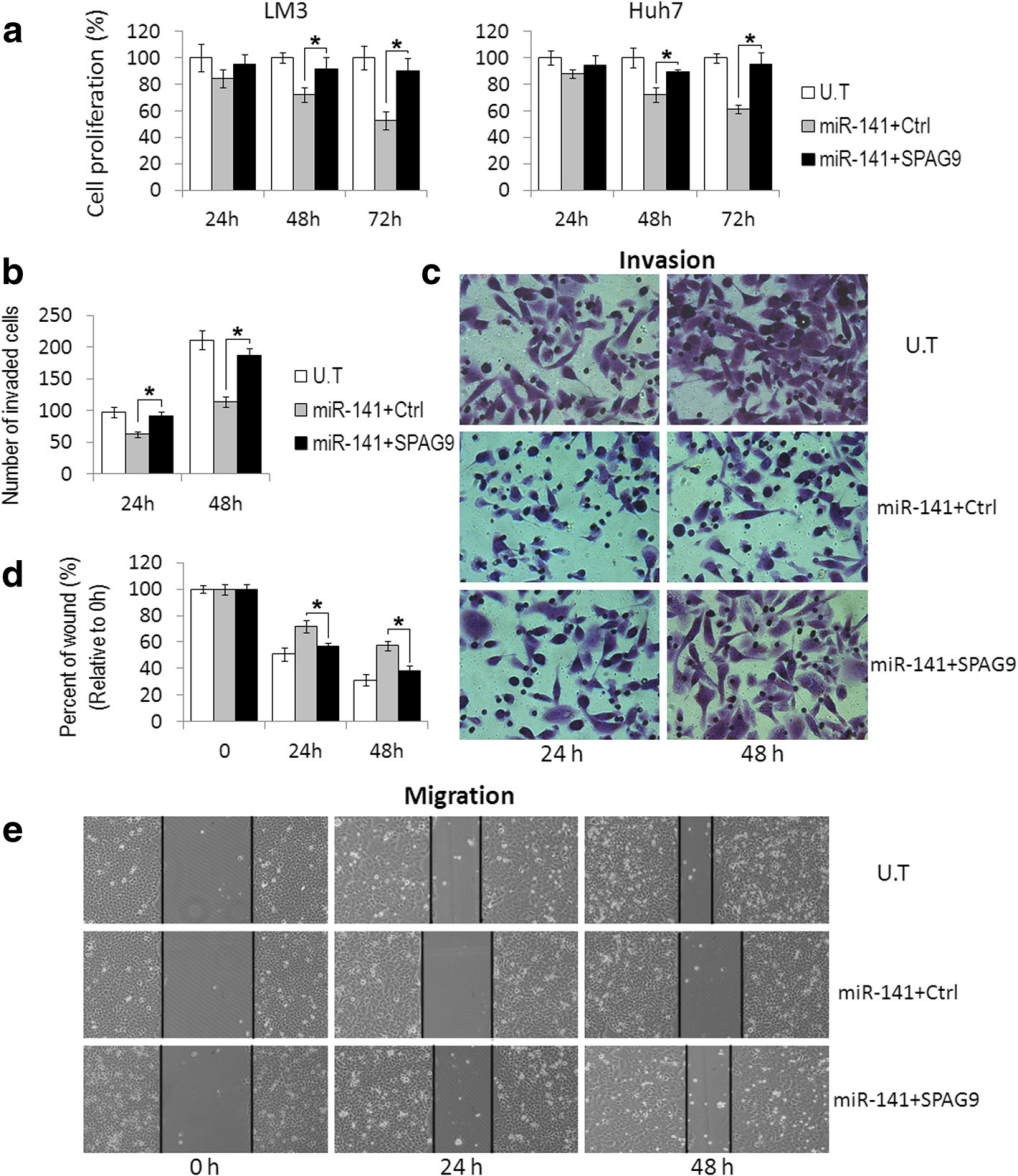
MiR-141 inhibits HCC cell growth and metastasis by targeting SPAG9. **a** SPAG9 plasmid or control vector was transfected into miR-141-overexpression HCC cells. MTT assay showed that the restoration of SPAG9 in miR-141-transfected cells attenuated the inhibitory effects of miR-141 on cell proliferation, especially at 48 h and 72 h. **b**, **c** Matrigel invasion assay showed that restoration of SPAG9 reversed the inhibitory effects of miR-141 on the invasion of Huh7 cells. **d**, **e** Wound-healing assay showed that the restoration of SPAG9 increased the migration of miR-141-transfected Huh7 cells. Data are presented as the mean ± S.D. (*P < 0.05, n = 3). U.T: untreated HCC cells; miR-141 + Ctrl: miR-141 and control vector co-transfected HCC cells; miR-141 + SPAG9: miR-141 and SPAG9 plasmid co-transfected HCC cells

Furthermore, to determine whether miR-141 represses cell proliferation and metastasis by decreasing SPAG9 level, we transduced either a control vector or a SPAG9 overexpression plasmid into HCC cells combined with miR-141 transfection. We found that the overexpression of SPAG9 attenuated the inhibitory effects of miR-141 mimics on HCC cell proliferation (Fig. 4a). Wound-healing and transwell assay also showed that the restoration of SPAG9 reversed the inhibitory effects of miR-141 mimics on HCC cell migration and invasion, respectively (Fig. 4b-e).

Overall, these results indicate that miR-141 might modulate cell proliferation and metastasis by targeting SPAG9.

### MiR-141 regulates JNK signaling pathway via SPAG9 in HCC cells

**Fig. 5:**
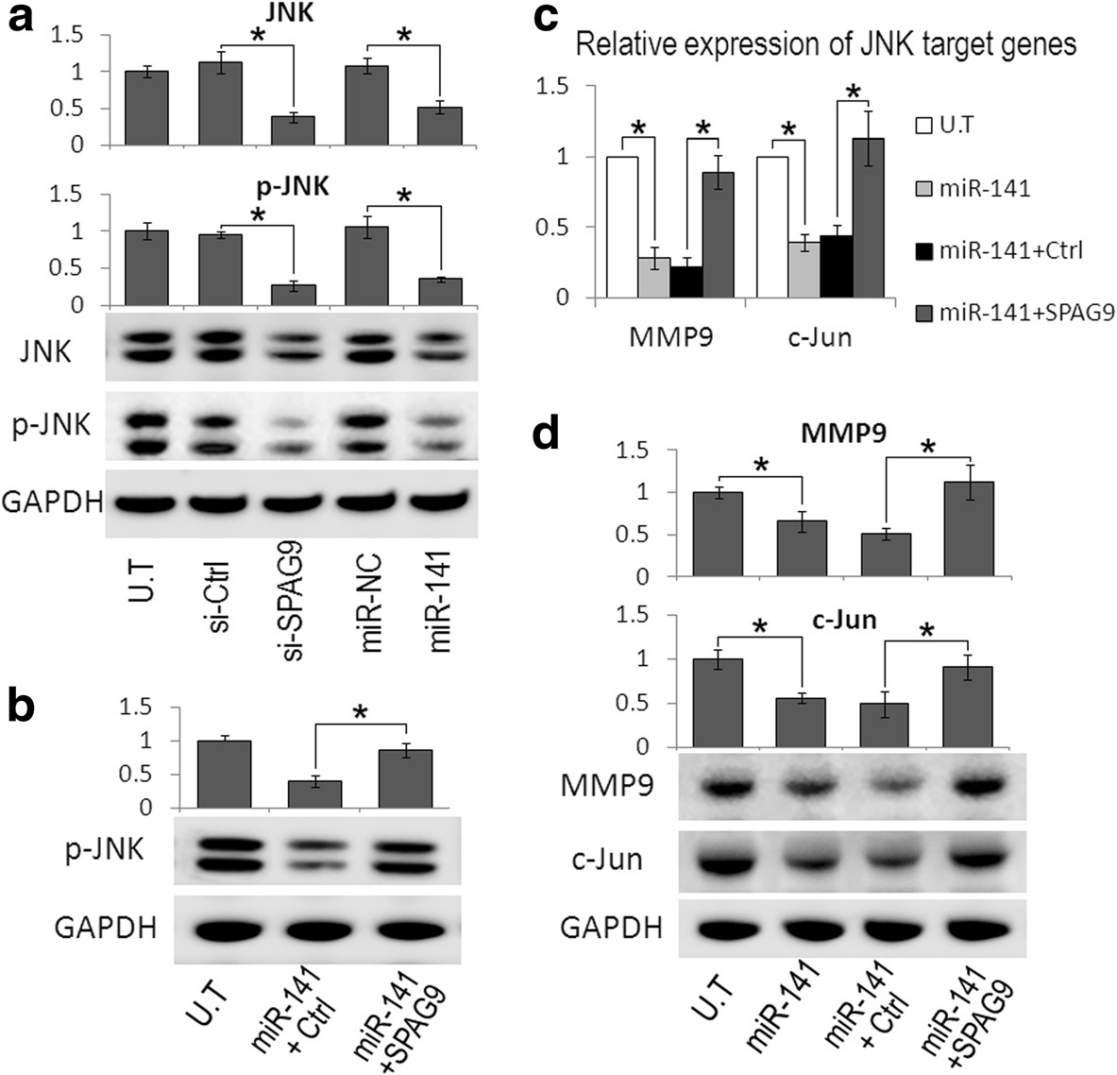
MiR-141 regulates JNK signaling pathway via SPAG9 in HCC cells. **a** Western blot analysis showed that SPAG9 depletion or miR-141 transfection decreased JNK protein expression and downregulated JNK phosphorylation in Huh7 cells. **b** Western blot analysis showed that SPAG9 overexpression significantly restored the expression of phosphorylated JNK in miR-141-transfected Huh7 cells. **c** and **d** Real time-PCR (**c**) and Western blot analysis (**d**) of MMP9 and c-Jun expression in Huh7 cells transfected with miR-141 or co-transfected with miR-141 and SPAG9 plasmid. Data are presented as the mean ± S.D. (*P < 0.05, n = 3). U.T: untreated Huh7 cells; miR-141 + Ctrl: miR-141 and control vector co-transfected Huh7 cells; miR-141 + SPAG9: miR-141 and SPAG9 plasmid co-transfected Huh7 cells

SPAG9 was reported to be involved in JNK signaling activation [3], which is closely related to the occurrence and development of HCC. To provide further mechanistic insight into the role of miR-141 and SPAG9 in HCC progression, we evaluated the effect of miR-141 on JNK activation. We found that JNK and p-JNK decreased in Huh7 cells after the transfection with miR-141 mimics or SPAG9 siRNA (Fig. 5a). To further determine if miR-141 repressed the JNK activity via SPAG9, we transfected miR-141 combined with SPAG9 overexpression in HCC cells. Figure 5b shows that the SPAG9 overexpression significantly restored JNK activity in miR-141-transfected Huh7 cells. We also quantified the JNK downstream target gene levels after miR-141 overexpression, such as MMP9 and c-Jun. We discovered that the upregulation of miR-141 dramatically decreased c-Jun and MMP9 expression, while the restoration of SPAG9 expression reversed the effects of miR-141 on genes expression (Fig. 5c and d). Overall, these data suggest that miR-141 inhibits the JNK signaling pathway via SPAG9.

## Discussion

SPAG9 is involved in the JNK-signaling module and play an important regulatory role in several physiologic and pathological processes, including cell survival, proliferation, apoptosis, and tumor development [23–25]. Previous studies have shown that the expression of SPAG9 was elevated in a variety of malignancies and correlated with tumor stage and prognosis, including HCC [4–8, 11]. At present, the role of aberrant-expressed SPAG9 in tumor development has been partly understood, while overexpressed SPAG9 may induce the activation of JNK signaling leading to tumor progression [4]. However, the underlying mechanism leading to SPAG9 overexpression is not well known. In this study, we identified miR-141 as a negative regulator of SPAG9 in HCC tumorigenesis.

MiR-141 was involved in the miR-200 family, which was known as a family of tumor suppressor miRNAs. The miR-200 family, which consists of five members (miR-200a, miR-200b, miR-200c, miR-429, and miR-141), are involved in the inhibition of epithelial-to-mesenchymal transition (EMT), repression of cancer stem cells (CSCs) self-renewal and differentiation, modulation of cell division and apoptosis, and reversal of chemoresistance [26, 27]. According to the prediction results of the algorithms, there are seed sequences of all five members in the 3′-UTR of the SPAG9 gene. Thus, we analyzed whether the miR-200 family could directly target SPAG9 3′-UTR with dual-luciferase reporter assay. All five members were found to suppress reporter gene activity in HEK293T cells; among them, miR-141 and miR-200a suppressed the reporter gene activity more significantly (Additional file 1: Figure S1A). We then assessed whether the ectopic expression of these five miRNAs mimicked the effects of SPAG9 interfering in HCC cells. As expected, ectopic overexpression of miR-200 s elicited the suppression of SPAG9 in Huh7 cells, especially miR-141 and miR-200a (Additional file 1: Figure S1B). Furthermore, the miR-141 and miR-200a expression in HCC tissues were investigated to verify their expression patterns with SPAG9. This analysis revealed that 9 out of 10 HCCs (90 %) had decreased miR-141 and increased SPAG9 expression, as compared with corresponding non-cancerous hepatic tissues, while 7 of 10 HCCs (70 %) had reduced miR-200a expression (Additional file 1: Figure S1C). The inverse correlation between SPAG9 expression and miR-141 was more prominent than that between SPAG9 and miR-200a. We further expand the sample size by analyzing the expression levels of SPAG9 and miR-141 in 24 other matched HCC and hepatic tissues. Additional file 1: Figure S2 shows that the SPAG9 expression was significantly inversely correlated with the miR-141 level in these HCC tissues. Moreover, the miRNA array analysis also presented that the expression levels of miR-200 family members, especially miR-141, were downregulated in a high SPAG9 expression HCC tissue or cell line as compared with low SPAG9 expression ones (Additional file 1: Figure S3). These results suggested that miR-141 is the most likely candidate miRNA regulating SPAG9 expression in liver tumorigenesis.

MiR-141 is negatively regulated in different types of cancers and is considered to be a tumor suppressor, e.g., ovarian cancer, breast cancer, or gastric cancer, by targeting a number of important genes such as p38α [28], Stat5a [29], TAZ [30], and others [31, 32]. Moreover, several in vivo studies have shown that the overexpression of miR-141 could suppress tumor growth and metastasis in a tumor xenograft mice model [30, 33]. In HCC, miR-141 was reported to be significantly down-regulated in human HCC tissues compared with adjacent normal tissues [34], and a low miR-141 expression was a substantial prognostic factor for the poor overall survival in HCC patients [35]. In the present study, we also found that the downregulation of miR-141 in HCC and the expression patterns of SPAG9 were inversely correlated with those of miR-141 in HCC tumor samples and cell lines (Fig. 1). In addition, the ectopic expression of miR-141 could decrease SPAG9 protein levels in HCC cells by directly targeting the 3′-UTR of SPAG9 mRNA (Fig. 2). Recently, several studies showed that miR-141 inhibited HCC growth, migration, and invasion by targeting the hepatocyte nuclear factor-3β (HNF-3β) [34], T lymphoma invasion and metastasis 1 (Tiam1) [35], and zinc finger E-box binding homeobox 2 (ZEB2) [36]. In the present study, we further expanded the function of miR-141 in HCC. We found that the upregulation of miR-141 could suppress HCC cell growth and invasion and knockdown of SPAG9-induced effects that were similar to those stimulated by miR-141 (Fig. 3). To further confirm that SPAG9 is a functional target of miR-141, we transfected miR-141 overexpressing HCC cells with SPAG9 plasmid, which encrypted the full-length coding sequence of SPAG9 without its 3′-UTR and found that the overexpression of SPAG9 lacking its 3′-UTR substantially reversed the tumor-suppressive effects of miR-141 (Fig. 3). These results demonstrate that SPAG9 is another functional target gene of miR-141 in HCC, and miR-141 may also elicit a tumor-suppressing effect via SPAG9 targeting.

Previous research demonstrated that SPAG9 was engaged in JNK pathway [37], which is closely related to tumor progression. To further provide mechanistic insight into the role of miR-141 and SPAG9 in HCC growth and metastasis, we examined the effect of miR-141-mediated SPAG9 regulation on JNK activation. We found that the p-JNK level was reduced by miR-141 overexpression, similar to those by SPAG9 silencing (Fig. 5a). Moreover, the overexpression of SPAG9 lacking it’s 3′-UTR significantly restored JNK activity in miR-141-transfected Huh7 cells (Fig. 5b), indicating that miR-141 activates the JNK signaling pathway via SPAG9 regulation. Since c-Jun and MMP9, which are a major downstream molecule and a target gene in the JNK pathway, were reported to play important roles in JNK-mediated tumor growth and metastasis [38], we further examined the effect of miR-141 and SPAG9 on these gene expressions. As anticipated, the overexpression of miR-141 significantly decreased c-Jun and MMP9 expression, while the restoration of SPAG9 expression reversed the effects of miR-141 on these genes (Fig. 5c and d). Previous research also showed that SPAG9 might act as an important promoter of tumor invasion via the SPAG9/JNK/MMP9 pathway in lung cancer [4]. MiR-141 is reported to function as a tumor suppressor and inhibit EMT and metastasis of tumor cells by targeting ZEB2 [39, 40]. Our study also showed that the overexpression of miR-141 could downregulate the protein level of ZEB2 (data not shown). Thus, the anti-metastasis effect of miR-141 via targeting the EMT regulator ZEB2 could not be partly excluded. However, Fig. 4 shows that the restoration of SPAG9 expression in miR-141-transfected cells significantly reversed the inhibitory effects of miR-141 on the migration and invasion of HCC cells, indicating that the SPAG9-JNK-MMP9 axis is also engaged in the anti-metastasis effect of miR-141 on HCC.

## Conclusions

Our results suggest that the downregulation of miR-141 may cause an aberrant overexpression of SPAG9 in human HCC. In addition, our data also expanded the function of tumor-suppressive miR-141. MiR-141-mediated suppression of SPAG9 inhibits cell growth and metastasis through the JNK signaling pathway, thereby providing novel therapeutic options for HCC.

## Ethics approval and consent to participate

This study was approved by the ethics committee of First Affiliated Hospital of Zhejiang University, and informed consent was obtained from each patients.

## Additional file

Additional file 1:
**Supplemental material.** (PDF 369 kb)

## Abbreviations

SPAG9: sperm-associated antigen 9
HCC: hepatocellular carcinoma
JNK: c-Jun NH2-terminal kinase
CT: cancer testis
MTT: 3-(4,5-dimethyl-2-thiazolyl)-2,5-diphenyl-2-H-tetrazolium bromide
T: HCC tissues
N: adjacent non-cancerous liver tissues
SPAG9-WT: wild-type SPAG9 3′-UTR reporter vector
SPAG9-Mut: mutant SPAG9 3′-UTR reporter vector
MiR-NC: non-target control miRNA
U.T: untreated
Si-Ctrl: control siRNA
Si-SPAG9: SPAG9 siRNA
MMP9: matrix metalloprotein 9
EMT: epithelial-to-mesenchymal transition
CSCs: cancer stem cells
HNF-3β: hepatocyte nuclear factor-3β
Tiam1: T lymphoma invasion and metastasis 1
ZEB2: zinc finger E-box binding homeobox 2

## References

[1] El-Serag HB (2011). Hepatocellular carcinoma. N Engl J Med.

[2] Giordano S, Columbano A (2013). MicroRNAs: new tools for diagnosis, prognosis and therapy in HCC?. Hepatology.

[3] Jagadish N, Rana R, Selvi R, Mishra D, Garg M, Yadav S (2005). Characterization of a novel human sperm-associated antigen 9 (SPAG9) having structural homology with c-Jun N-terminal kinase-interacting protein. Biochem J.

[4] Wang Y, Dong Q, Miao Y, Fu L, Lin X, Wang E (2013). Clinical significance and biological roles of SPAG9 overexpression in non-small cell lung cancer. Lung Cancer.

[5] Kanojia D, Garg M, Gupta S, Gupta A, Suri A (2009). Sperm-associated antigen 9, a novel biomarker for early detection of breast cancer. Cancer Epidemiol Biomarkers Prev.

[6] Garg M, Kanojia D, Suri S, Gupta S, Gupta A, Suri A (2009). Sperm-associated antigen 9: a novel diagnostic marker for thyroid cancer. J Clin Endocrinol Metab.

[7] Garg M, Kanojia D, Salhan S, Suri S, Gupta A, Lohiya NK (2009). Sperm-associated antigen 9 is a biomarker for early cervical carcinoma. Cancer.

[8] Kanojia D, Garg M, Gupta S, Gupta A, Suri A (2011). Sperm-associated antigen 9 is a novel biomarker for colorectal cancer and is involved in tumor growth and tumorigenicity. Am J Pathol.

[9] Sinha A, Agarwal S, Parashar D, Verma A, Saini S, Jagadish N (2013). Down regulation of SPAG9 reduces growth and invasive potential of triple-negative breast cancer cells: possible implications in targeted therapy. J Exp Clin Cancer Res.

[10] Chen F, Lu Z, Deng J, Han X, Bai J, Liu Q (2014). SPAG9 expression is increased in human prostate cancer and promotes cell motility, invasion and angiogenesis in vitro. Oncol Rep.

[11] Xie C, Fu L, Liu N, Li Q (2014). Overexpression of SPAG9 correlates with poor prognosis and tumor progression in hepatocellular carcinoma. Tumour Biol.

[12] Dp B (2004). MicroRNAs Genomics, Biogenesis, Mechanism, and Function. Cell.

[13] Wang Z, Yao H, Lin S, Zhu X, Shen Z, Lu G (2013). Transcriptional and epigenetic regulation of human microRNAs. Cancer Lett.

[14] Liu X, Chen X, Yu X, Tao Y, Bode AM, Dong Z (2013). Regulation of microRNAs by epigenetics and their interplay involved in cancer. J Exp Clin Cancer Res.

[15] Yang N, Ekanem NR, Sakyi CA, Ray SD (2015). Hepatocellular carcinoma and microRNA: new perspectives on therapeutics and diagnostics. Adv Drug Deliv Rev.

[16] Fan MQ, Huang CB, Gu Y, Xiao Y, Sheng JX, Zhong L (2013). Decrease expression of microRNA-20a promotes cancer cell proliferation and predicts poor survival of hepatocellular carcinoma. J Exp Clin Cancer Res.

[17] Wang B, Hsu SH, Wang X, Kutay H, Bid HK, Yu J (2014). Reciprocal regulation of microRNA-122 and c-Myc in hepatocellular cancer: role of E2F1 and transcription factor dimerization partner 2. Hepatology.

[18] Cheng Z, Wang HZ, Li X, Wu Z, Han Y, Li Y (2015). MicroRNA-184 inhibits cell proliferation and invasion, and specifically targets TNFAIP2 in Glioma. J Exp Clin Cancer Res.

[19] Zhang GJ, Li JS, Zhou H, Xiao HX, Li Y, Zhou T (2015). MicroRNA-106b promotes colorectal cancer cell migration and invasion by directly targeting DLC1. J Exp Clin Cancer Res.

[20] Zhou B, Chen H, Wei D, Kuang Y, Zhao X, Li G (2014). A novel miR-219-SMC4-JAK2/Stat3 regulatory pathway in human hepatocellular carcinoma. J Exp Clin Cancer Res.

[21] Kim HS, Lee KS, Bae HJ, Eun JW, Shen Q, Park SJ (2015). MicroRNA-31 functions as a tumor suppressor by regulating cell cycle and epithelial-mesenchymal transition regulatory proteins in liver cancer. Oncotarget.

[22] Ni F, Zhao H, Cui H, Wu Z, Chen L, Hu Z (2015). MicroRNA-362-5p promotes tumor growth and metastasis by targeting CYLD in hepatocellular carcinoma. Cancer Lett.

[23] Garg M, Kanojia D, Khosla A, Dudha N, Sati S, Chaurasiya D (2008). Sperm-associated antigen 9 is associated with tumor growth, migration, and invasion in renal cell carcinoma. Cancer Res.

[24] Davies C, Tournier C (2012). Exploring the function of the JNK (c-Jun N-terminal kinase) signalling pathway in physiological and pathological processes to design novel therapeutic strategies. Biochem Soc Trans.

[25] Chen F (2012). JNK-induced apoptosis, compensatory growth, and cancer stem cells. Cancer Res.

[26] Feng X, Wang Z, Fillmore R, Xi Y (2014). MiR-200, a new star miRNA in human cancer. Cancer Lett.

[27] Humphries B, Yang C (2015). The microRNA-200 family: small molecules with novel roles in cancer development, progression and therapy. Oncotarget.

[28] Mateescu B, Batista L, Cardon M, Gruosso T, De Feraudy Y, Mariani O (2011). miR-141 and miR-200a act on ovarian tumorigenesis by controlling oxidative stress response. Nat Med.

[29] Finlay-Schultz J, Cittelly DM, Hendricks P, Patel P, Kabos P, Jacobsen BM (2015). Progesterone downregulation of miR-141 contributes to expansion of stem-like breast cancer cells through maintenance of progesterone receptor and Stat5a. Oncogene.

[30] Zuo QF, Zhang R, Li BS, Zhao YL, Zhuang Y, Yu T (2015). MicroRNA-141 inhibits tumor growth and metastasis in gastric cancer by directly targeting transcriptional co-activator with PDZ-binding motif, TAZ. Cell Death Dis.

[31] Chiyomaru T, Fukuhara S, Saini S, Majid S, Deng G, Shahryari V (2014). Long non-coding RNA HOTAIR is targeted and regulated by miR-141 in human cancer cells. J Biol Chem.

[32] Zhao G, Wang B, Liu Y, Zhang JG, Deng SC, Qin Q (2013). miRNA-141, downregulated in pancreatic cancer, inhibits cell proliferation and invasion by directly targeting MAP4K4. Mol Cancer Ther.

[33] Chen X, Wang X, Ruan A, Han W, Zhao Y, Lu X (2014). miR-141 is a key regulator of renal cell carcinoma proliferation and metastasis by controlling EphA2 expression. Clin Cancer Res.

[34] Lin L, Liang H, Wang Y, Yin X, Hu Y, Huang J (2014). microRNA-141 inhibits cell proliferation and invasion and promotes apoptosis by targeting hepatocyte nuclear factor-3β in hepatocellular carcinoma cells. BMC Cancer.

[35] Liu Y, Ding Y, Huang J, Wang S, Ni W, Guan J (2014). MiR-141 suppresses the migration and invasion of HCC cells by targeting Tiam1. PLoS One.

[36] Wu SM, Ai HW, Zhang DY, Han XQ, Pan Q, Luo FL (2014). MiR-141 targets ZEB2 to suppress HCC progression. Tumour Biol.

[37] Ando K, Uemura K, Kuzuya A, Maesako M, Asada-Utsugi M, Kubota M (2011). N-cadherin regulates p38 MAPK signaling via association with JNK-associated leucine zipper protein: implications for neurodegeneration in Alzheimer disease. J Biol Chem.

[38] Rong Z, Li L, Fei F, Luo L, Qu Y (2013). Combined treatment of glibenclamide and CoCl2 decreases MMP9 expression and inhibits growth in highly metastatic breast cancer. J Exp Clin Cancer Res.

[39] Du Y, Wang L, Wu H, Zhang Y, Wang K, Wu D (2015). MicroRNA-141 inhibits migration of gastric cancer by targeting zinc finger E-box-binding homeobox 2. Mol Med Rep.

[40] Roy SS, Gonugunta VK, Bandyopadhyay A, Rao MK, Goodall GJ, Sun LZ (2014). Significance of PELP1/HDAC2/miR-200 regulatory network in EMT and metastasis of breast cancer. Oncogene.

