# Supplementary material for: Direct targeting sperm-associated antigen 9 by miR-141 influences hepatocellular carcinoma cell growth and metastasis via JNK pathway
Source: J Exp Clin Cancer Res. 2016 Jan 21;35:14. doi: 10.1186/s13046-016-0289-z (PMC4721207; doi:10.1186/s13046-016-0289-z)
Supplement: Additional file 1: — Supplemental material. (PDF 369 kb) [file 13046_2016_289_MOESM1_ESM.pdf]

Supplemental material:

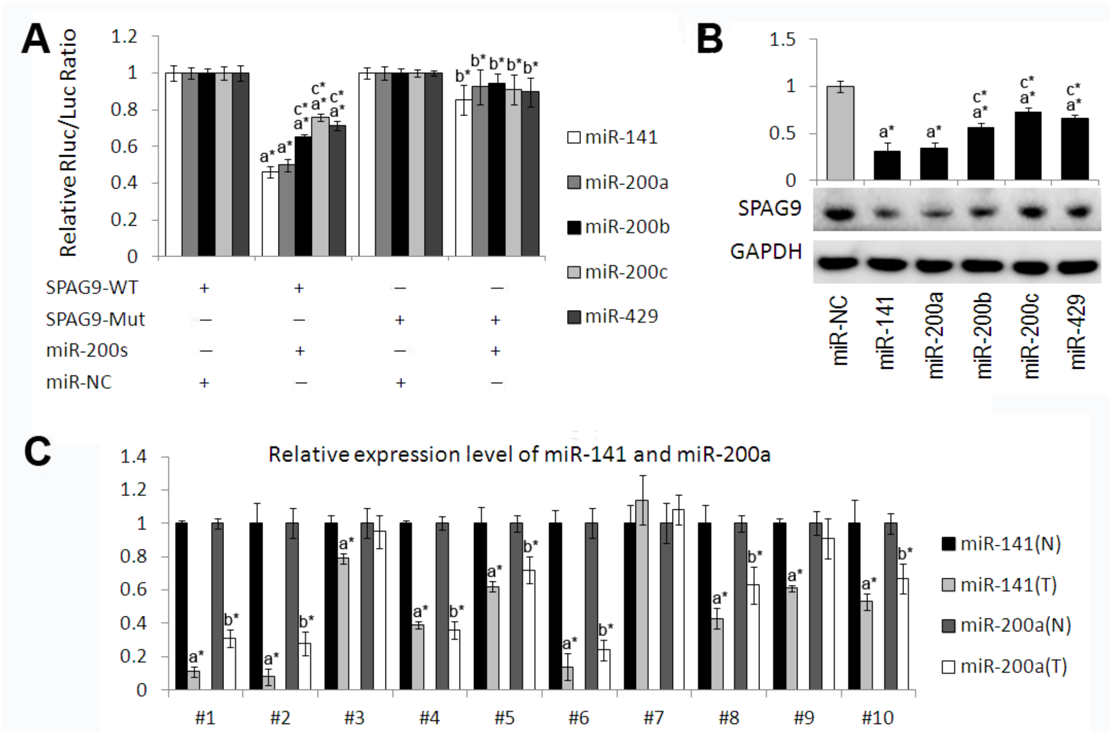

Suppl. Fig. 1 Identification of miR-200 family targeting SPAG9 in human HCC

(A) Dual-luciferase activity of the wild-type (WT) and mutant (Mut) SPAG9 3’-UTR reporter constructs in the presence of miR-200 family members (miR-141, miR-200a, miR-200b, miR-200c, and miR-429) or control miRNA (miR-NC). *T*-test of wild-type SPAG9 3’-UTR constructs activity in Huh7 cells by transfection with miR-200s versus miR-NC (a) or transfection with miR-141 versus other miR-200s (c); b: *T*-test of relative luciferase activity of wild-type SPAG9 3’-UTR constructs versus those of mutant constructs by transfection with miR-200s. (B) Western blot analysis of SPAG9 expression in Huh7 cells transfected with miR-200 family members or control miRNA (miR-NC). *T*-test of SPAG9 expression levels in Huh7 cells transfection with miR-200s versus miR-NC (a) or transfection with miR-141 versus other miR-200s (c). (C) Real time-PCR analysis of relative miR-141 and miR-200a levels in 10 paired HCC (T) and non-cancerous hepatic tissues (N). *T*-test of miR-141 (a) or miR-200a (b) levels in HCC versus non-cancerous hepatic tissues. Data is presented as the mean ±S.D (*$P < 0.05$, n=3).

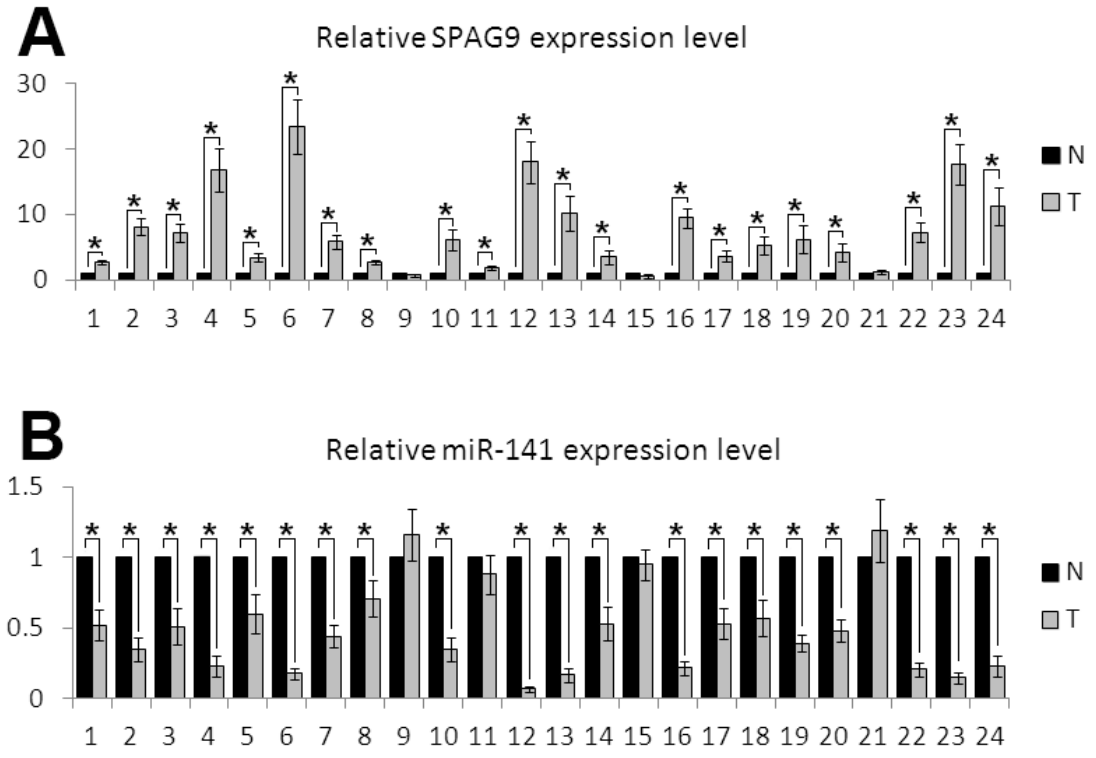

Suppl. Fig. 2 The expression levels of SPAG9 and miR-141 in HCC tissues

(A) The protein level of SPAG9 in 24 paired HCC (T) and non-cancerous hepatic tissues (N). (B) The expression level of miR-141 in 24 paired HCC (T) and non-cancerous hepatic tissues (N). Data is presented as mean ±S.D. P values were calculated using the t-test, $*P < 0.05$.

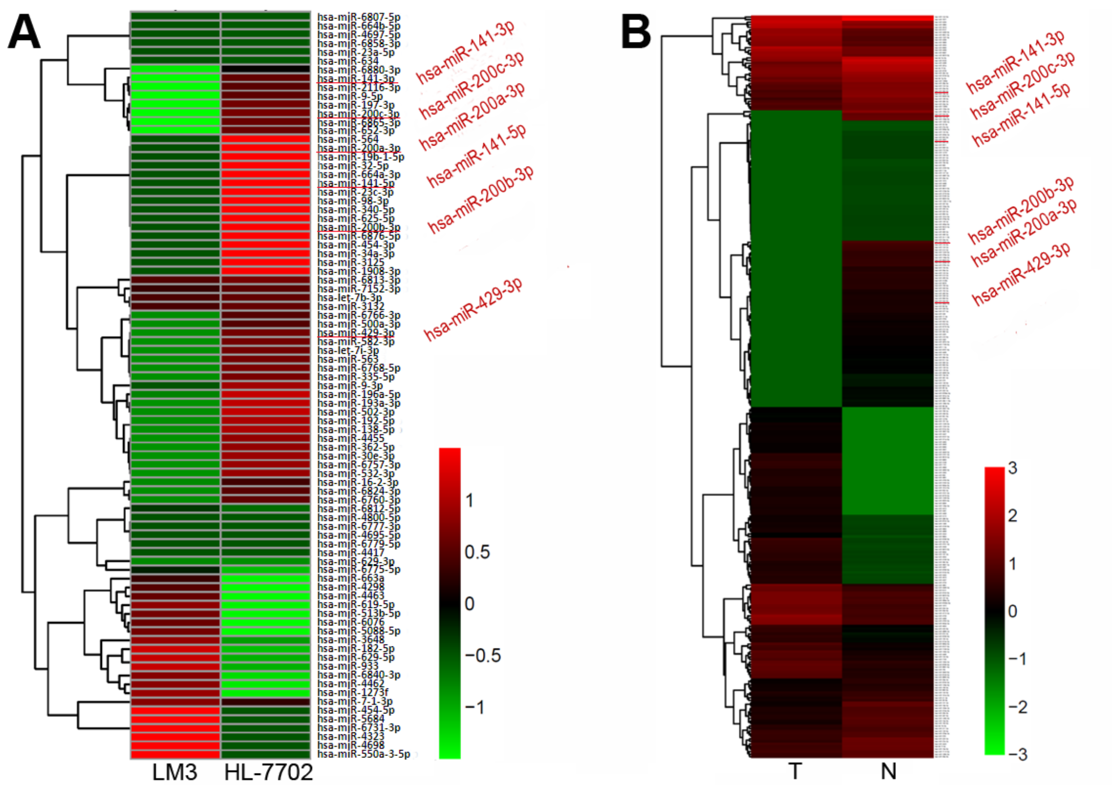

Suppl. Fig. 3 miRNA array analysis on HCC tissue and cell lines

Agilent human miRNA Microarrays were used for miRNA array analysis on HCC tissue and cell lines. (A) Heatmap illustrating miRNomes profiles for LM3 (high SPAG9 expression) and HL-7702 (low SPAG9 expression) cell lines. (B) Heatmap illustrating miRNomes profiles for matched HCC (T) and non-cancerous hepatic tissues (N).
